# Spectrum of clinical and radiographic findings in patients with diagnosis of H1N1 and correlation with clinical severity

**DOI:** 10.1186/s12879-019-4592-0

**Published:** 2019-11-12

**Authors:** Karla Schoen, Natally Horvat, Nicolau F. C. Guerreiro, Isac de Castro, Karina S. de Giassi

**Affiliations:** 1 Department of Radiology, Hospital Sírio-Libanês, Rua Dona Adma Jafet, 91, Bela Vista, São Paulo, SP 01308-050 Brazil; 2 Department of Epidemiology and Biostatistics, Hospital Sírio-Libanês, São Paulo, Brazil

**Keywords:** Influenza, H1N1, Infection, Radiology, Outcome

## Abstract

**Background:**

The aim of this study was to evaluate the correlation between clinical and imaging findings with a worse clinical outcome in patients with a confirmed diagnosis of H1N1 influenza A virus.

**Methods:**

Patients with a positive viral test for influenza A H1N1 in 2016 and chest radiography (CR) and/or computed tomography (CT) results had clinical and imaging data reviewed. Hospitalization, admission to the intensive care unit or death were defined as worse clinical outcomes. The association between clinical and imaging features and the worse outcome was calculated in a logistical regression model.

**Results:**

Eighty of 160 (50%) patients were men, with a mean age of 43 ± 19 years. The most common symptoms were as follows: flu-like symptoms 141/160 (88%), dyspnea (25/160, 17%), and thoracic pain (7/160, 5%). Abnormalities on CR were detected in 8/110 (7%) patients, and 43/59 (73%) patients had an abnormal CT. The following variables were associated with worse clinical outcomes: the presence of *diabetes mellitus* (DM), hypertension, dyspnea, thoracic pain, abnormal CR or CT regardless of the type of finding, CT with consolidation or ground glass opacity.

**Conclusions:**

The presence of DM, hypertension, dyspnea, thoracic pain, or an abnormal CR or CT on admission were associated with worse clinical outcomes in patients with H1N1 influenza A virus infection. Thus, the use of readily accessible clinical and imaging features on admission may have a role in the evaluation of patients with H1N1 infection.

## Background

Influenza A H1N1 virus is the subtype of influenza virus that typically leads to a more severe infection than the usual seasonal influenza virus, and its viral strains were historically responsible for the major outbreaks worldwide, similar to the Spanish flu in the twentieth century. It was the cause of the last influenza pandemic in 2009, which resulted in thousands of deaths worldwide, principally among young individuals and pregnant woman. Since then, the H1N1 influenza virus has assumed a seasonal spreading similar to other influenza types and continues to cause substantial morbidity, although it is controlled with the immunization programs [1]. The immunization campaign for Influenza A H1N1 in our country recommends the vaccination for the entire population annually from April to May. The public healthcare system offers all year round the vaccine for risk groups, including pregnant women, elderly patients, children, healthcare professionals and people with any chronic disease. Nevertheless, an atypical outbreak of Influenza A H1N1 infection occurred in South America in 2016, which started before the vaccination campaign, and caused approximately 800 deaths and 500 intensive care unit admissions in a single country [2].

Since the first outbreak of H1N1, many studies have been conducted to identify an imaging pattern [3, 4] or clinical features that indicate a worse prognosis [1, 5, 6]. The identification of clinical and radiographic findings in patients with H1N1 infection that correlate with the clinical severity is of key importance in the management of these patients. Previous studies have demonstrated that all patients with H1N1 admitted to an intensive care unit had at least an abnormal conventional radiograph (CR) at diagnosis. However, there is a relative lack of studies in the literature that have evaluated predictive tools in the evaluation of patients with H1N1 [7, 8].

In this scenario, the purpose of this study was to evaluate the correlation between clinical and imaging findings with a worse clinical outcome in patients with a confirmed diagnosis of H1N1 influenza A virus.

## Methods

**Fig. 1:**
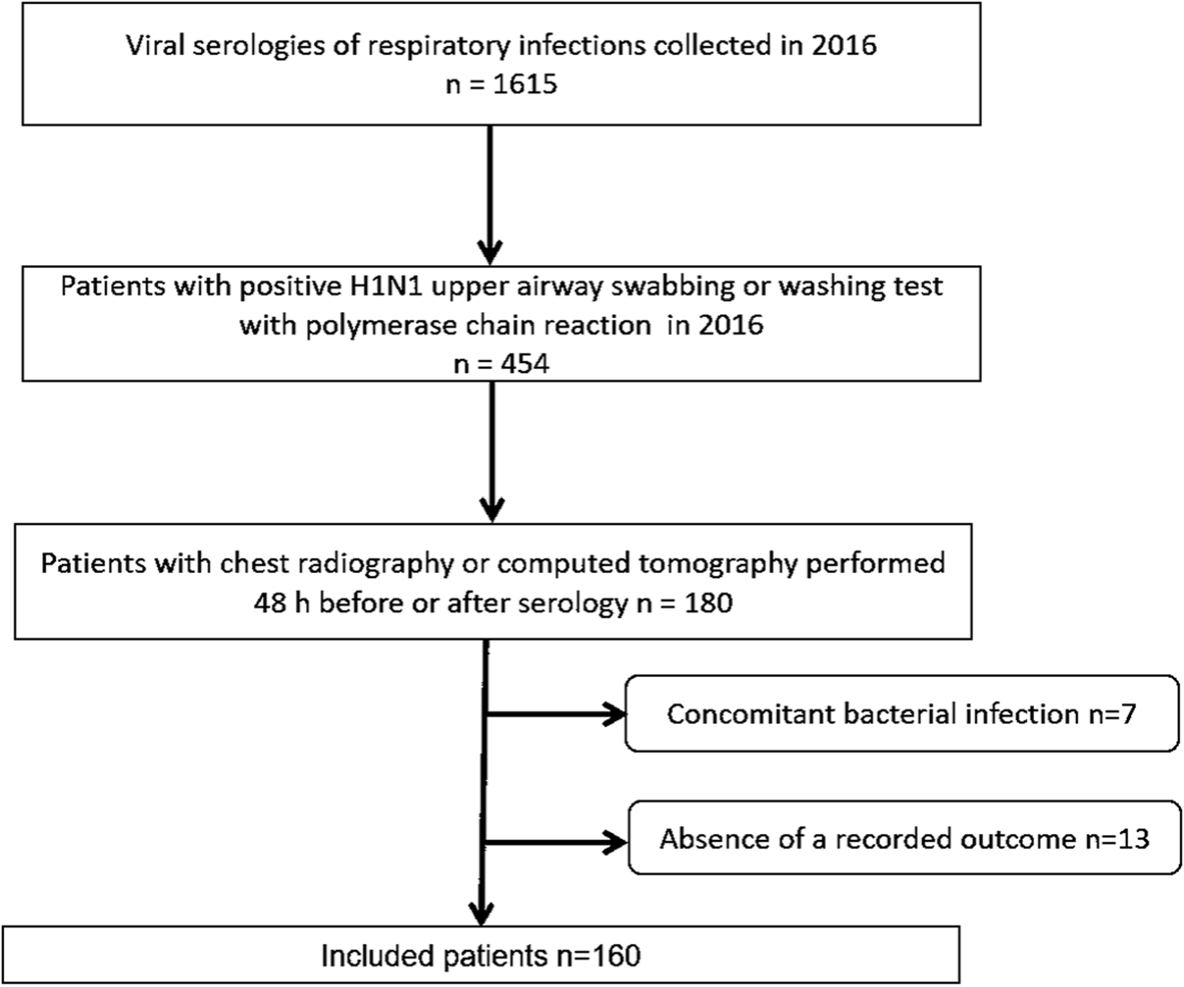
Flowchart of patient selection

The institutional review board approved this retrospective study and waived the requirement for informed consent. The database from our institution, which is a private hospital, was searched for consecutive patients with a diagnosis of influenza A H1N1 in 2016 as assessed via the upper airway by nasopharyngeal swabbing or a washing test with polymerase chain reaction. The inclusion criteria were a positive test for influenza A H1N1 and CR and/or chest computed tomography (CT) within 48 h before or after the positive test. The exclusion criteria were another confirmed concomitant pulmonary infection and the absence of follow-up data in the medical records. The patient accrual is summarized in Fig. 1.

Clinical and laboratorial data were obtained from a detailed medical record review conducted by two radiologists with 2 and 3 years of experience, respectively, using a standardized form. The following clinical data of the patients were assessed: gender, age, symptoms at admission (such as flu-like symptoms, dyspnea, thoracic pain, hemoptysis, and sepsis), and presence of comorbidities (including systemic hypertension, *diabetes mellitus* [DM], tobacco smoke, asthma, heart disease, chronic obstructive pulmonary disease [COPD], immunodeficiency, and other). Information regarding the physical examination at admission was also evaluated, including the heart rate (HR), temperature, oxygen saturation (O_2_), and blood pressure (BP). Regarding the laboratory data, the white blood count, lymphocytes, and C-reactive protein (CRP) obtained at admission were assessed.

The chest CR were performed in a digital CR device in the posteroanterior and lateral incidences during maximal inspiration.

CT scans were performed on two of the following devices: a dual-source 256 row or a 128 row detector CT system. Contrast imaging was performed with 1.0–1.5 ml/kg injected at a flow rate of 4.0–5.5 ml/s^− 1^. Images were reconstructed in the axial view using a slice thickness of 1 mm and an increment of 0.7 mm.

**Fig. 2:**
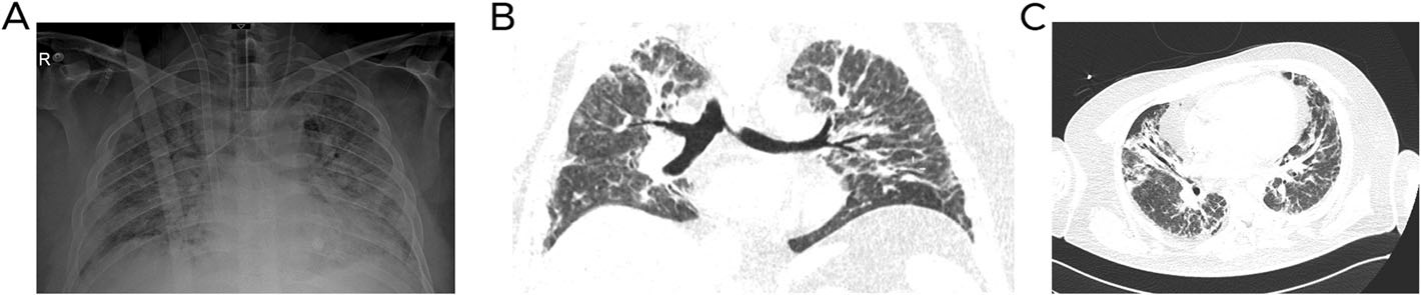
Forty-five-year-old man without comorbidities with H1N1 virus infection who was admitted to the intensive care unit with acute respiratory distress syndrome. **a** Anteroposterior chest radiography demonstrates bilateral diffuse pulmonary infiltrates and consolidations. **b** and **c** Chest computed tomography on coronal and axial planes also show consolidations and bilateral ground-glass opacities

**Fig. 3:**
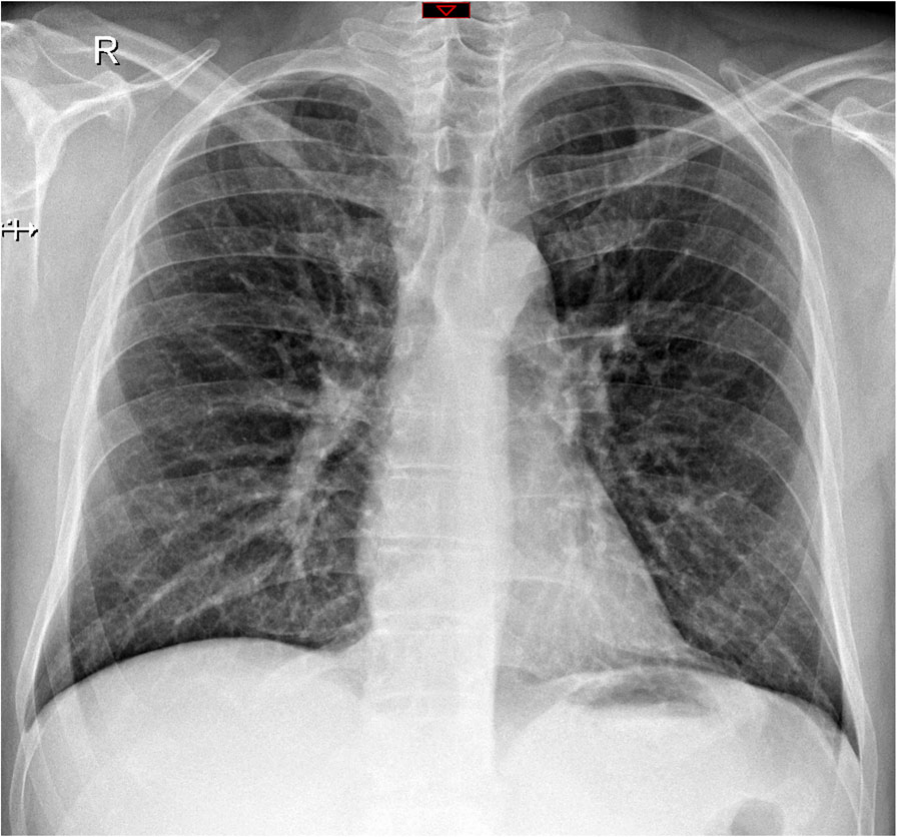
Sixty-five-year-old woman without comorbidities with H1N1 infection who did not have a worse outcome. Normal chest radiography performed at emergency department

**Fig. 4:**
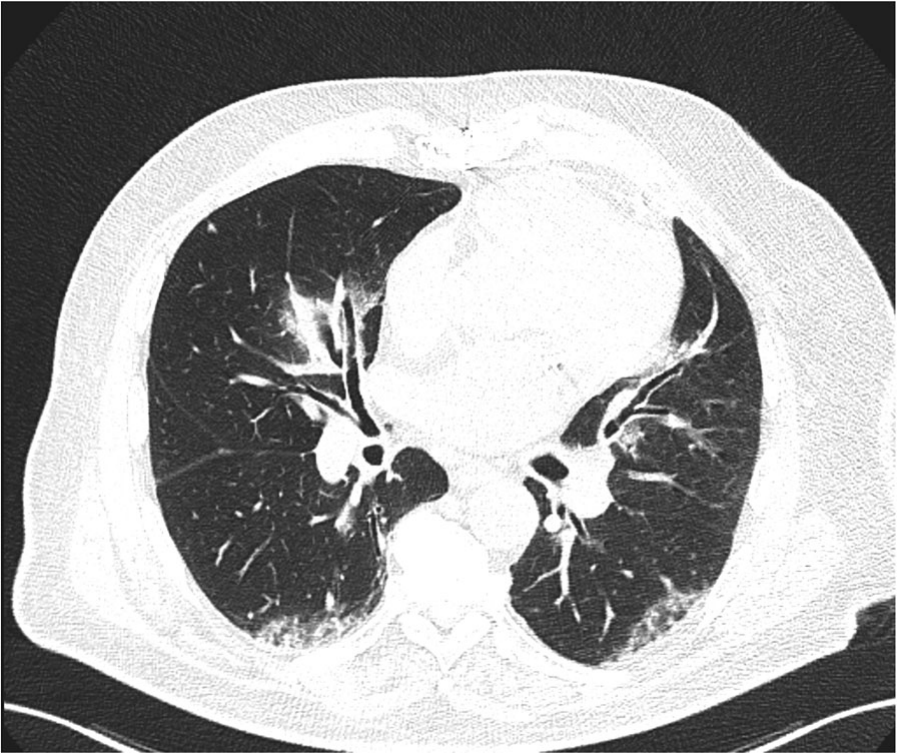
Sixty-nine-year-old man admitted at the intensive care unit with dyspnea. Axial chest computed tomography demonstrates ground-glass opacities with peribronchovascular distribution

Two board-certificated chest radiologists (with 6 and 2 years of experience) blinded to the clinical data independently reviewed the radiological. The radiologists analyzed the imaging features according to the Fleischner Society glossary [9]. For discordant imaging findings (6/146 images; 5%), an agreement was reached by consensus between the readers. Figures 2, 3 and 4 summarize the imaging features assessed by the radiologists.

The following imaging features were evaluated on CR: pulmonary infiltrate (which was defined as any alterations, including both ground glass and reticular opacities), consolidation, and pleural effusion [10, 11]. The CT imaging features were fully assessed and the following findings were highlighted: ground glass opacity (GGO), consolidation, bronchial wall thickening, septal interlobular thickening, lymph node enlargement, tree-in-bud pattern, and pleural effusion. The distribution of the features was defined as follows: none, 1 field, 2 fields unilateral, 3 or more fields bilateral, and diffuse.

The patients were classified into two groups: good clinical outcome and worse clinical outcome. A good clinical outcome was defined as hospital discharge after evaluation in the emergency department without the need for hospitalization. Patients who were hospitalized, admitted to the intensive care unit (ICU) or died were determined to have a worse clinical outcome.

### Statistical analysis

Continuous variables were tested for normality with the Kolmogorov-Smimov and Shapiro Wilk tests. The values are expressed as median and percentiles 25 and 75 or as the mean and standard deviation for parametric and non-parametric data respectively. The categorical data are presented as absolute values and percentages and were tested using Pearson χ2 test and Fisher exact test, if applicable. The Mc’Nemar test was used by compare two methods. Non-parametric data was compared using the Mann-Whitney U test for two independent samples. The logistic regression models included calculations of corresponding crude and adjusted odds ratios (ORs) and 95% confidence intervals (CI). Two-tailed *p*-values ≤0.05 were considered significant. Statistical analyses were carried out using SPSS for Windows version 19.0 (Chicago, IL, USA). Hierarchical cluster analysis was performed to identify individual similarities in the diagnostic model through analysis of clusters to try to determine from each of the groups to which they belong. Statistical significance was considered with *p* ≤ 0,05. Analyses were performed using SPSS 19.0 and GraphPad Prism 5.0.

## Results

Our study population consisted of 160 patients, including 80/160 (50%) men and 80/160 (50%) women, with a median age of 43 years (IQR: 32–56). None of the patients were children or pregnant. Forty-nine/160 (30.6%) patients had comorbidities, 12/160 (7.5%) patients had DM, 29/160 (18.1%) patients had systemic hypertension, 4/160 (2.5%) patients had COPD, 6/160 (3.8%) patients had another pneumopathy and 8/160 (5.0%) patients had immunosuppression. None of the patients had history of heart disease or other chronic disease. Five of 160 (3.1%) patients were smokers. None of the patients had asthma. The vast majority of the included patients (147/160, 91.8%) were diagnosed in March and April, followed by 6/160 (3.7%) in May, 2/160 (1.3%) in February, 2/160 (1.3%) in June, 1/160 (0.6%) in July, and 2/160 (1.3%) in August.

With regards to the clinical manifestations at admission, 144 patients had this information written in the medical records. Based on this subpopulation, 141/144 (97.9%) patients had flu-like symptoms, 25/144 (17.4%) patients had dyspnea, and 7/144 patients had thoracic pain. None of the patients were septic or had hemoptysis. On physical examination, the median oxygen saturation was 96% (IQR: 95–98), the median HR was 92 bpm (IQR: 80–107), 49/141 (34.7%) patients had high temperature levels, with a median temperature of 37 °C (IQR: 36.3–37.7), the median systolic BP was 125.5 mmHg (IQR: 113–138), and the median diastolic BP was 78.5 mmHg (IQR: 69–87). Regarding the laboratory data, the median white blood cell count and lymphocytes were 5990/mm^3^ (IQR: 4610–7560) and 950/mm^3^ (IQR: 680–1400), respectively, and the median of CRP was 1.90 mg/dL (IQR: 0.96–3.88).

Considering outcome, 37/160 (23.1%) patients were hospitalized and classified as having a worse clinical outcome. Four of these patients (4/37, 10.8%) required intensive care unit (ICU) admission, and none of these patients died. The mean age of this subpopulation was 50 years old (IQR: 34–66). All patients who were hospitalized had an abnormality on CR and/or CT.

Table 1 summarizes the patients’ characteristics and the radiological findings.

**Table 1:** Patient characteristics

Characteristics	All patients (*n* = 160)	Good clinical outcome (*n* = 123)	Worse clinical outcome (*n* = 37)	P*
Clinical Data				
Gender M/F	80 (50.0) / 80 (50.0)	65 (52.8) / 58 (47.2)	15 (40.5) / 22 (40.5)	0.189
Age (years), median (IQR)	43 (32–56)	42 (32–54)	52 (35–69)	0.027
Flu-like symptoms^a^	141 (97.9)	104 (97.2)	37 (100)	0.303
Dyspnea^a^	25 (17.4)	13 (12.1)	12 (32.4)	0.005
Thoracic pain^a^	7 (4.9)	2 (1.9)	5 (13.5)	0.005
Comorbidities^a^	49 (30.6)	33 (26.8)	16 (43.2)	0.058
*Diabetes mellitus*^a^	12 (7.5)	5 (4.1)	7 (18.9)	0.003
Systemic hypertension^a^	29 (18.1)	17 (13.8)	12 (32.4)	0.010
Tobacco smoke^a^	5 (3.1)	5 (4.1)	0 (0)	0.213
Chronic obstructive pulmonary disease^a^	4 (2.5)	3 (2.4)	1 (2.7)	0.928
Immunodeficiency^a^	8 (5)	6 (4.9)	2 (5.4)	0.897
Cardiac frequency (bpm), median (IQR)	92 (80–107)	93 (82–108)	86 (75–103)	0.087
Temperature (°C), median (IQR)	37 (36.3–37.7)	37.4 (36.7–37.8)	36.4 (36–37)	< 0.001
Oxygen saturation (%), median (IQR)	96 (95–98)	96 (96–98)	96 (95–98)	0.173
Systolic blood pressure (mmHg), median (IQR)	125.5 (113–138)	128 (113.5–139)	120 (110–130)	0.203
Diastolic blood pressure (mmHg), median (IQR)	78.5 (69–87)	78.5 (69.5–89)	77.5 (68–80)	0.157
Laboratorial Data				
White blood count (/mm^3^), median (IQR)	5990 (4610–7260)	5990 (4610–7250)	6055 (4545–8310)	0.683
Lymphocytes (/mm^3^), median (IQR)	950 (680–1400)	890 (680–1270)	985 (680–1540)	0.335
C-reactive protein (mg/dL), median (IQR)	1.90 (0.96–3.88)	1.75 (0.87–3.67)	3.05 (1.56–7.50)	0.026
Radiological Data				
Abnormal CR^b^	8 (7.1)	4 (4)	4 (28.6)	0.001
CR – Pulmonary infiltrate^b^	3 (2,7)	1 (1)	2 (14.3)	0.004
CR – Consolidation^b^	2 (1.8)	0 (0)	2 (14.3)	< 0.001
CR – Pleural effusion^b^	1 (0.9)	1 (0)	1 (7.1)	0.008
Abnormal CT^c^	43 (71.7)	17 (56.7)	26 (86.7)	0.010
CT – Ground glass opacity^c^	27 (45)	9 (30)	18 (60)	0.020
CT – Consolidation^c^	9 (15)	1 (3.3)	8 (26.7)	0.011
CT – Bronchial thickening^c^	26 (43.3)	14 (46.7)	12 (40.0)	0.602
CT – Pleural effusion^c^	5 (8.3)	2 (6.7)	3 (10.0)	0.640

Note: except where otherwise indicated, data represent number (%). *CR* Chest radiography, *CT* Computed tomography, *IQR* Interquartile range. * Univariate analysis. ^a^144 cases available; ^b^113 cases available; ^c^60 cases available

One hundred thirteen patients underwent CR, and 8/113 (7.1%) of them were abnormal. Pulmonary infiltrate was detected in 5/8 (62.5%) patients, consolidation was identified in 2/8 (25.0%) patients and pleural effusion was found in 1/8 (12.5%) patients.

Sixty patients underwent chest CT, and 43/60 (71.7%) patients showed some abnormality. Twenty-seven/60 (45.0%) patients had GGO, 9/60 (15%) patients had consolidation, 26/60 (43.3%) patients had bronchial thickening, and 5/60 (8.3%) patients had pleural effusion. No other CT findings such as septal interlobular thickening, lymph node enlargement or tree-in-bud pattern were found.

Thirteen patients underwent both CR and chest CT (Table 2). There was a significant correlation between an abnormal CR and abnormal chest CT (*p* = 0.031) and between the presence of pulmonary infiltrate on CR and GGO on CT (*p* = 0.016). In contrast, there was no significant correlation between consolidation and pleural effusion on CR and CT (*p* = 0.501 and *p* = 0.999, respectively).

**Table 2:** Intertest agreement of chest X-ray and chest computed tomography

Chest X-ray	Chest CT		*P*
Abnormality	No	Yes	
No	4	6	0.031
Yes	0	3	
Pulmonary infiltrate / Ground-glass opacity			
No	5	7	0.016
Yes	0	1	
Pulmonary consolidation			
No	10	2	0.501
Yes	0	1	
Pleural effusion			
No	12	0	0.999
Yes	0	1	

### Correlation with worse clinical outcome

Considering the clinical data, the presence of DM, systemic hypertension, dyspnea, thoracic pain, temperature levels and CRP levels were significantly different between the groups (Table 1). The frequency of DM (7/37, 18.9% vs 5/123, 4,1%; *p* = 0.003), systemic hypertension (12/37, 32.4% vs 17/106, 13.8%; *p* = 0.010), dyspnea (12/25, 32.4% vs 13/107, 12.1%; *p* = 0.005), and thoracic pain (5/37, 13.5% vs 2/107, 1.9%; p = 0.005) were significantly higher in the patients who required hospitalization. The temperature was significantly lower in the patients with worse clinical outcomes (36.4 °C vs 37.4 °C, respectively, *p* < 0.001), and the C-reactive protein levels were significantly higher (3.05 vs 1.75, respectively, *p* = 0.026).

Regarding the imaging features on CR, the presence of an abnormality, pulmonary infiltrate, consolidation, and pleural effusion were significantly more frequent in the patients with worse outcomes ([4/14, 28.6% vs 4/99, 4%; *p* = 0.001], [2/14, 14.3% vs 1/99, 1%; *p* = 0.004], [2/14, 14.3% vs 0/99, 0.0%; p < 0.001], and [1/14, 7.1% vs 0/99, 0%; *p* = 0.008], respectively).

With regards to the imaging features on CT, the presence of an abnormality, GGO and consolidation were significantly more frequent in the patients with worse outcomes ([26/30, 86.7% vs 17/30, 56.7%; *p* = 0.010], [18/30, 60% vs 9/30, 30%; *p* = 0.020], and [8/30, 26.7% vs 1/30, 3.3%; *p* = 0.011]). Furthermore, the distribution of the imaging features on CR and CT were correlated with outcome, in which a diffuse pattern was more related to worse clinical outcomes.

In the logistic regression, the following data predicted worse outcomes: DM (OR: 8.3; 95% CI 1.4–50.2, p = 0.020), body temperature (OR: 0.4; 95% CI 0.3–0.8, *p* = 0.005), thoracic pain (OR: 20.3; 95% CI 2.2- *p* < 0.001), alteration on CR or CT (OR: 22.7, p < 0.001), pulmonary infiltrate on CR or GGO on CT (OR: 6.9, *p* = 0.002), and pleural effusion on CT or CR (OR: 10.3, *p* = 0.045).

Figures 2, 3 and 4 show patients with and without worse clinical outcomes.

**Fig. 5:**
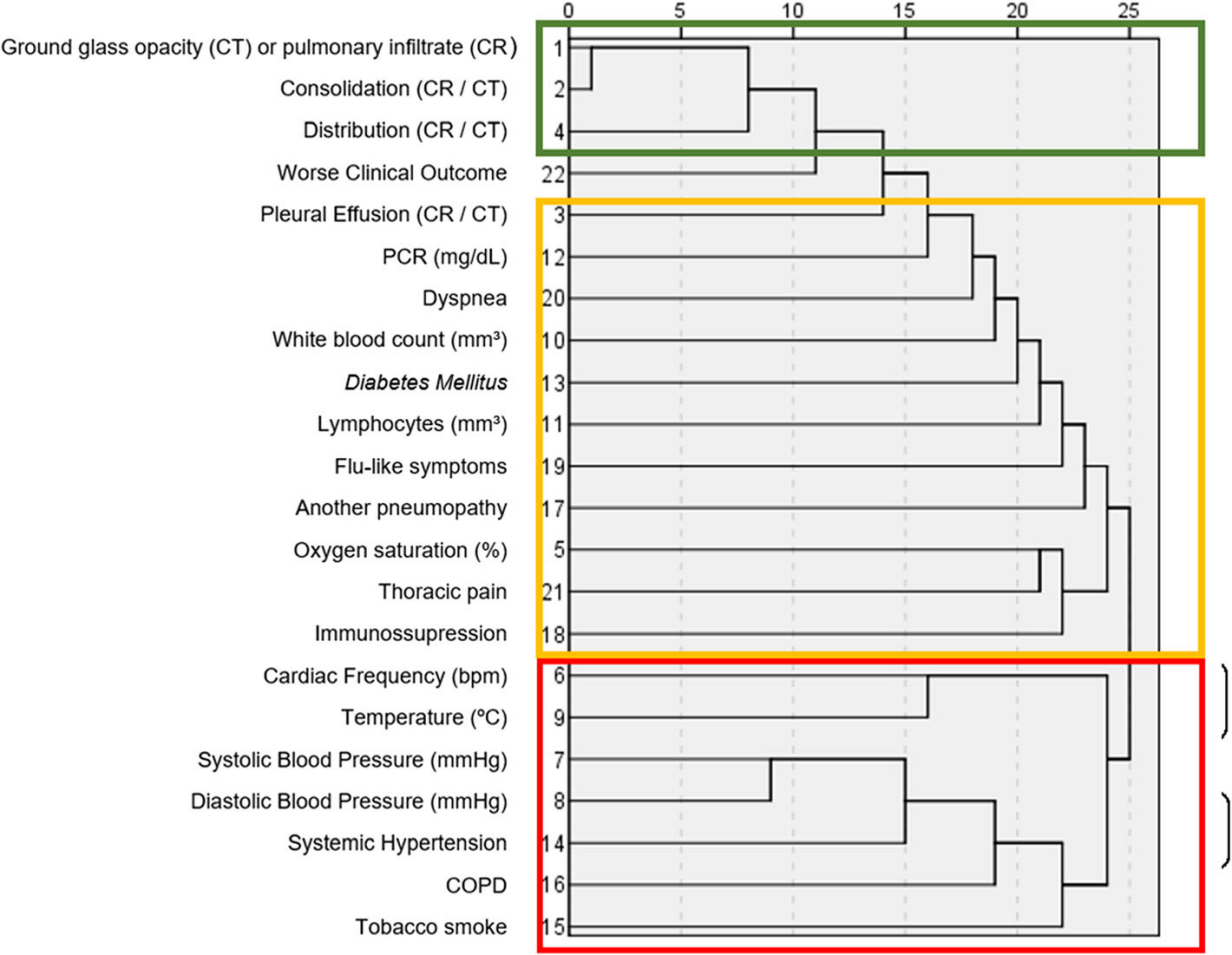
Dendrogram showing hierarchical clustering of variables in predicting worse clinical outcomes. The variables with higher correlations with worse clinical outcomes are highlighted in green, with moderate correlations in yellow and variables with low correlations in red

In the hierarchical cluster analysis, three clusters were identified (Fig. 5). The cluster that better correlated with worse clinical outcomes contained the following variables: GGO on CT or CR, consolidation on CT or pulmonary infiltrate on CR and the extension of the disease.

## Discussion

Our study population was obtained from our database throughout the year of 2016; however, all patients selected had the H1N1 infection diagnosed between the 3 months before winter in the south hemisphere, which is unusual of a typical seasonal respiratory infection and configured an atypical and early outbreak of H1N1 infection [12].

The major clinical findings among our population were flu-like symptoms (97.9%) as expected for a respiratory infection [13]. We found in our population clinical and radiological changes associated with worse clinical outcomes. DM, systemic hypertension, dyspnea, thoracic pain, temperature and CRP levels were the clinical features associated with worse clinical outcomes. Moreover, the radiological findings were the presence of an abnormality on CT or CR, pulmonary infiltrate, consolidation, and pleural effusion on CR, presence of GGO and consolidation on CT, and diffuse pattern of imaging abnormality on CT or CR. In the logistic regression, the presence of DM, thoracic pain, levels of body temperature, an alteration on CR or CT, diffuse pattern of distribution of any abnormality on CR or CT; pulmonary infiltrate on CR, GGO on CT, and pleural effusion on CT or CR were the variables that predicted worse clinical outcomes. In the hierarchical cluster analysis, the cluster that better correlated with worse clinical outcomes contained GGO on CT, pulmonary infiltrate on CR, consolidation and the diffuse pattern of distribution on CT or CR.

Regarding the clinical features, our results are in line with similar studies that evaluated symptoms associated with a worse outcome, as they demonstrated that fever and dyspnea may predict hospitalization [13]. Studies that have analyzed only patients with severe H1N1 admitted to an ICU have also demonstrated that symptoms were similar to typical seasonal H1N1, such as cough, dyspnea, fever, myalgia and headache [1]. The results are also comparable to studies that have analyzed mild and severe infections [7].

Our study also demonstrated chest pain as a predictor of worse outcome, a feature that was not found in previous studies. However, another study also identified particularities that may be related to the institution sample or type of H1N1 virus. Rohani et al. [1] found gastrointestinal symptoms associated with severe infection with a worse outcome. Therefore, despite minor differences among studies, H1N1 infection exhibits similar clinical features that predict severity over the years regardless of the genotype. In contrast to previous studies, we did not identify death due to H1N1 infection. The average mortality among adult patients in the United States was 26% [14]. Our study also demonstrated that body temperature was significantly lower in patients with worse clinical outcomes. Although the underlying reason of hypothermia in these patients remains unclear, some other studies also have demonstrated an association between low body temperature and worse outcomes in critically ill patients with and without infection [15, 16].

With regards to imaging features, the CR and CT findings in our study population are comparable to the most representative studies over the years since the first H1N1 outbreak in 2009 [5, 13, 17]. All 37 patients with worse outcomes had at least a pulmonary abnormality in an imaging exam, and we identified significant differences among the groups with and without hospitalization.

The imaging features detected in our population are in line with previous data of viral pneumonia, including pulmonary infiltrate, GGO, consolidation and pleural effusion [10, 18]. Comparable to other studies, we found that diffuse distribution on an imaging abnormality was associated with worse clinical outcomes [5, 13, 17].

The clinical relevance of our results essentially relies on the management of patients with H1N1 infection. Informing the multidisciplinary team of the potential for a worse clinical outcome based on easily accessible information, such as the presence of DM, thoracic pain, body temperature, and an abnormality on CR or CT, may add value in the setting of emergency departments. If clinicians know in advance the possibility of a worse outcome, they may consider a closer evaluation, and the emergency discharge decision may be postponed. This type of guidance may be valuable, particularly at noncomprehensive centers and during epidemic situations.

There are several potential limitations of this study. First, it is a single-center retrospective study performed at a private hospital focused on adult non-pregnant patients, which is thus subjected to selection bias. Second, the study population seemed to have a milder infection than previous studies, without deaths or sepsis at admission. These results may be due to an earlier outbreak of 2016 in the early autumn or due to a less severe H1N1 genotype. Although our study population was relatively large compared to other imaging studies, future prospective multicenter studies with a more diverse population, including pediatric and pregnant patients are needed to overcome the limitations of this study and to provide a better generalization of our results.

## Conclusions

In conclusion, our study performed at a private hospital focused on adult non-pregnant patients demonstrates that the presence of DM, hypertension, dyspnea, thoracic pain, or an abnormal CR or CT on admission were associated with worse clinical outcomes in patients with H1N1 influenza A virus infection. Consolidation and ground glass opacities on CT were also associated with the clinical severity in these patients. Thus, the use of readily accessible clinical and imaging features on admission may have a role in the evaluation of patients with H1N1 infection.

## Data Availability

The datasets generated and/or analyzed during the current study are not publicly available due medical confidentiality, the research was made through the medical records on our institution database, but are available from the corresponding author on reasonable request.

## Abbreviations

BP: Blood pressure
CI: Confidence intervals
COPD: Chronic obstructive coronary disease
CR: Conventional radiograph
CRP: C-reactive protein (CRP)
CT: Computed tomography
*DM*: Diabetes mellitus
GGO: Ground glass opacity (GGO)
HR: Heart rate (HR)
ICU: Intensive care unit
OR: Odds ratio
